# Hippocampal CA3 activation alleviates fMRI-BOLD responses in the rat prefrontal cortex induced by electrical VTA stimulation

**DOI:** 10.1371/journal.pone.0172926

**Published:** 2017-02-27

**Authors:** Thomas Scherf, Frank Angenstein

**Affiliations:** 1 Functional Neuroimaging Group, Deutsches Zentrum für neurodegenerative Erkrankungen (DZNE), Magdeburg, Germany; 2 Leibniz Institute for Neurobiology, Magdeburg, Germany; University of North Carolina at Chapel Hill, UNITED STATES

## Abstract

Functional magnetic resonance imaging (fMRI) was used to identify brain- wide networks that are activated by electrical stimulation of either the ventral tegmental area (VTA) or hippocampal CA3 region. Stimulation of either one of these regions caused significant BOLD responses in common structures, such as the septum and left and right hippocampus, but also in unique structures, such as the medial prefrontal cortex region/anterior cingulum region (mPFC/ACC) and striatum, which were only activated during VTA stimulation. Concurrent stimulations of the two structures resulted in no additive BOLD responses but significantly reduced BOLD responses in the mPFC/ACC when compared with sole VTA stimulation. This reduction is caused by costimulation of the hippocampal CA3 region, which was itself not sufficient to modify BOLD signal intensities in the mPFC/ACC. Under this experimental condition, functional connectivity between VTA and mPFC/ACC in terms of neurophysiological interactions was causative, driven by direct electrical stimulation of VTA projecting neurons, the resulting functional connectivity in terms of correlated BOLD time series becoming masked as soon as hippocampal projections concurrently coactivated mPFC neurons. This result warns against misinterpretation of the absence of functional connectivity in fMRI data sets, because strong existing neurophysiological interactions can be obscured by unrelated network activities.

## Introduction

Functional magnetic resonance imaging (fMRI) visualizes with high spatial resolution local changes in hemodynamics, i.e., variations in blood flow, blood volume and/or blood oxygenation, which in turn are controlled by changes in neuronal activity. Although the underlying neuro-vascular coupling mechanisms are still not completely understood, fMRI has already become a valued tool to detect synchronized neuronal activities and thus, to map brain-wide neuronal networks. The rationale for an fMRI approach to mapping functionally connected regions in the brain arises from the assumption that as soon as one region drives via direct connections the activity in another region, the resulting fMRI signal intensities in these regions will develop in a similar manner, i.e., the BOLD (blood oxygen level dependent) time series in these regions correlate. This however, presumes that variations in BOLD signal intensities are always directly related to the amount of incoming activity. There are several evidences that the time course of BOLD signal intensities in one particular brain region is not only controlled by the incoming activity from one specific brain region but also critically depends on the quality of local neuronal network activity [1–3]. This implies that BOLD-fMRI may fail to detect existing functional connectivities, as soon as concurrently incoming inputs from other brain regions substantially alter the quality of local network activity.

A straightforward experimental approach to test this assumption is to electrically stimulate one brain region and detect stimulus-related BOLD signal intensity changes in all brain regions that receive (monosynaptically) projections from the activated region, i.e., visualize functional connectivities of the stimulated region. Because the application of biphasic electrical pulses will directly stimulate projecting neurons, the existing functional connectivity only depends on the responsiveness of the neurons in the target region. To address the question of how reliable BOLD-fMRI detects existing functional connectivities under different conditions, we modified this experimental approach and activated separately and/or concurrently two partially overlapping brain-wide neuronal networks. First, the left hippocampal CA3 region was electrically activated, which has previously be shown to induce significant BOLD responses in the left and right hippocampus and septum [4] and potentially in the prefrontal cortex [5] and second, the ventral tegmental area (VTA) was electrically activated, which has previously been shown to cause the formation of significant BOLD responses in the VTA, septum, prefrontal cortex, and parts of the hippocampus [6]. The VTA is linked with a number of brain regions, such as the nucleus accumbens (NAcc), medial prefrontal cortex (mPFC/ACC), hippocampus, amygdala, and septum [7–10].

Under this experimental condition, the applied electrical pulses should always drive existing functional connectivities; thus, as long one structure is stimulated in an identical manner, the functional connectivities to its target regions should remain similar, independently of putative changes in the activities of other brain structures. That means, if BOLD-fMRI is a reliable measure of existing functional connectivities, correlations of BOLD time series between the two stimulated regions and one target region should either remain similar when the target region receives only one input or even additive when the target region receives concurrent inputs from the two stimulated structures.

## Material and methods

### Animals and surgical procedure

Animals were cared for and used according to a protocol approved by the animal experiment committee and in conformity with the European convention for the protection of vertebrate animals used for experimental purposes and institutional guidelines 86/609/CEE, November 24, 1986. The experiments were approved by the animal care committee of the State Saxony-Anhalt, Germany (No. 203.h-42502-2-1218DZNE) and were performed according to the ARRIVE (Animal Research: Reporting *In Vivo* Experiments) guidelines. For all experiments, 39 animals (experiment 1: n = 13; experiment 2: n = 11, experiment 3: n = 6, and experiment 4: n = 9) were scanned. Five animals (experiment 1: n = 2, experiment 2: n = 2, and experiment 4: n = 1) were not included in the final analysis because of motion artifacts (see Data Processing and Analysis).

**Fig 1:**
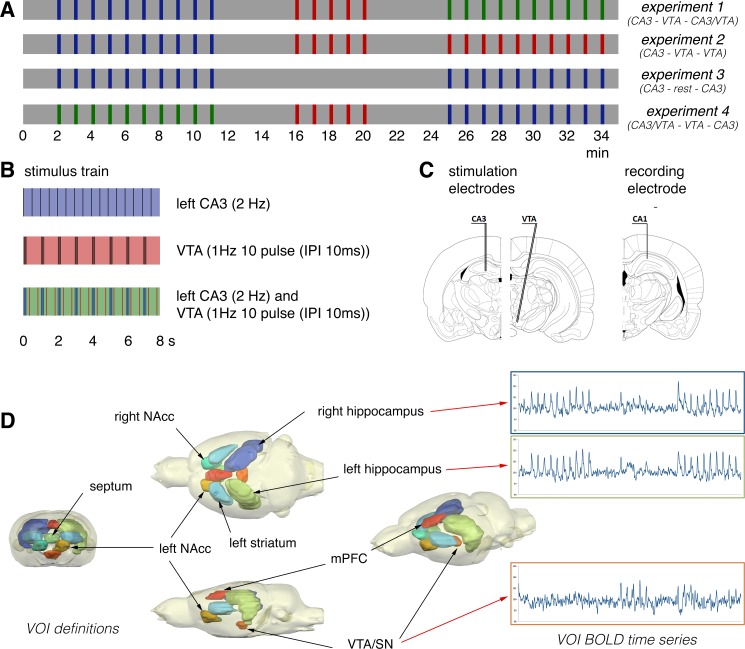
Summary of applied stimulation protocols and VOI analysis. (A) During one experiment, three consecutive stimulation blocks were applied, and every stimulation block consisted of identical stimulation trains. One stimulation train lasted 8 s followed by 52 s rest; thus, one train every minute. Colors indicate the forms of stimulation. blue: left CA3, red: VTA, and green: CA3 and VTA stimulation. (B) Summary of applied pulses per stimulation train. The left CA3 was stimulated with continuous 2-Hz pulses (indicated by black lines). VTA was stimulated with eight bursts of 10 pulses (inter-pulse interval 10 ms), one burst/s. For costimulation, the onset of continuous 2-Hz pulse CA3 stimulation was delayed by 200 ms to prevent stimulation artifacts from the VTA stimulation from interfering with the recording of field potentials in the CA1 region during low-frequency CA3 stimulations. (C) Location of stimulation and recording electrodes (see Material and Method section). (D) Location of analyzed VOIs (NAcc–nucleus accumbens, mPFC/ACC–medial prefrontal cortex/anterior cingulum). For each animal, BOLD time series of all voxels located in one VOI were averaged and depicted as BOLD time series of the appropriate VOI. For group analysis, VOI BOLD time series from all individual animals were averaged.

For electrode implantation, 9-week-old male Wistar rats were anesthetized with Nembutal (40 mg/kg i.p.) and placed in a stereotactic frame. One bipolar stimulation electrode (114 μm in diameter, made from Teflon-coated tungsten wire) was placed in the CA3 region at the coordinates AP: −1.8, ML: −1.4 mm from bregma, DV: 2.8 to 3.3 mm from the dural surface (n = 6). One monopolar recording electrode was lowered into the stratum radiatum of the CA1 at the coordinates AP: −4.0 mm, ML: 2.3 mm from bregma, DV: 2.1–2.6 mm from the dural surface (Fig 1). Correct placement was controlled by monitoring the monosynaptic-evoked field potentials during implantation, especially regarding electrode depth. A second bipolar stimulation electrode was placed in the ventral tegmental area (coordinates: AP -5.6 mm, ML +2.3 mm from bregma, DV 7.8 mm from the dural surface angled 10° to the midline). The correct placement of this stimulation electrode during implantation was verified by application of a short stimulation protocol (burst of 10 pulses with an inter-pulse interval of 10 ms, 300 μA stimulation intensity). Correct placement was assumed when stimulation in the VTA caused clear whisker movements. All coordinates for electrode implantation were taken from the atlas of Paxinos and Watson [11]. The exact locations of the electrodes were histologically verified after completion of the experiments. Grounding and indifferent electrodes (silver wires) were set on the dura through the right side of the skull and fixed in place using acrylic dental cement and plastic screws. The electrodes were additionally attached to a miniature plastic socket and fixed with acrylic dental cement. The wounds were treated with a chlorhexidine-containing medical powder. Following surgery, animals were provided with *ad libitum* food and water and housed individually for a recovery period of 1 week.

### Magnetic resonance imaging (MRI) measurements and CA3 stimulation

Rats were initially anesthetized with 1.0%–1.5% isoflurane (in 50:50 N_2_:O_2_, v:v), and connected to the stimulation and recording electrodes. After the animal was placed on the stereotactic platform, narcosis was switched to deep sedation by applying a medetomidine (Domitor, Pfizer GmbH, Karlsruhe, Germany) bolus of 50 μg/kg subcutaneously, and after 15 min, 100 μg/kg·h subcutaneously [12]. The animals were fixed using a head holder with a bite bar to reduce motion artifacts. Heating was provided from the ventral side (body temperature was measured in some animals before and after the fMRI session and the temperature remained stable between 37.5 and 38.5°C), and the breathing rate (between 40 and 60 breaths/min), heart rate (between 220 and 300 bpm) and oxygen saturation (between 97% and 99%) were monitored during the whole experiment using an MRI-compatible pulse oxymeter (Mouse Ox, Starr Life Sciences, Pittsburgh, PA, USA). Electrophysiological responses were evoked by a stimulus generator (Isolated Pulse Stimulator, Model 2100, Science Products, Hofheim, Germany) and recorded with a 5000-Hz sampling rate and pass filtered from 1 to 5 kHz using a quad channel differential extracellular amplifier (Ex4-400, Science Products, Hofheim, Germany). The signals were transformed by an analogue-to-digital interface (power-CED, Cambridge Electronic Design, Cambridge, U.K.) and stored on a personal computer. Because the switching gradients required for the acquisition of fMR-images only generated minor artifacts, no preprocessing of the recorded electrophysiological data was necessary. For each generated fEPSP, the slope (measured at the steepest rise of the first negative deflection in mv/ms) and the latency were determined. To calculate the appropriate stimulation intensities for the fMRI experiment, biphasic constant current pulses (pulse duration 0.2 ms) were applied to the hippocampal CA3 (30 min after switching to deep sedation by applying medetomidine) with increasing intensities (i.e., three test pulses at 10-s intervals for the following intensities: 50, 100, 200, 300, 400, 500, and 600 μA) and the evoked CA1 field potentials were recorded. The recordings were taken at 2-min intervals for lower intensities than 400 μA and 4-min intervals for all subsequent higher intensities. According to this input/output curve, the intensity required to evoke a fEPSP and the maximal measured slope function could be determined. The relation between the stimulation intensity and measured slope allows us to set the intensity for the stimulation of the CA3 region to a value that elicited approximately 80% of the maximal measured slope. This stimulation intensity also induced a spiking component inside the shape of the fEPSP. Electrical stimulation of the VTA at high intensities (i.e., >300 μA) caused eye and whisker movements, thus part of the measured animals could not be analyzed due to movement artifacts.

Magnetic resonance imaging experiments were performed on a Bruker Biospec 47/20 scanner (Bruker BioSpin GmbH, Ettlingen, Germany) at 4.7 T (free bore of 20 cm), equipped with a BGA09 (400 mT/m) gradient system (Bruker BioSpin GmbH, Ettlingen, Germany). A 50-mm Litzcage small animal imaging system (Doty Scientific, Columbia, SC, USA) was used for radio frequency (RF) excitation and signal reception. For anatomical images, eight horizontal T_2_-weighted spin-echo images were obtained using a rapid acquisition relaxation enhanced (RARE) sequence [13], configured using the following parameters: repetition time 4000 ms, echo time 15 ms, slice thickness 0.8 mm, field of view (FOV) 37 × 37 mm, matrix 256 × 256, RARE factor 8, averages 4. The total scanning time was 8 min 32 s. Functional MRI was performed using an EPI (echo planar imaging) sequence with the following parameters: repetition time 2000 ms, echo time 24 ms, slice thickness 0.8 mm, FOV 37 × 37 mm, matrix 92 × 92, and scanning time per frame 2 s.

For the combination of the fMRI and electrophysiological experiments, the stimulation protocol was divided into three stimulation blocks (Fig 1). Each stimulation block contained identical stimulation trains. Trigger pulses that were generated by the scanner at the beginning of every volume, i.e., every 2 s, were used to synchronize fMR-image acquisition and electrophysiological stimulations. The total time for the combined fMRI electrophysiology session was 35 min (1050 frames in fMRI).

### Data processing and analysis

Functional data were loaded and converted into BrainVoyager data format. A standard sequence of preprocessing steps implemented in the *BrainVoyager QX* software (Brain Innovation, Maastrich, the Netherlands), such as three-dimensional (3D) motion correction (trilinear interpolation and reduced data using the first volume as a reference) and temporal filtering [high pass (GLM-Fourier): 3 sines/cosines and Gaussian filter, FWHM 3 data points] were applied to each data set. Because the reconstruction of the fMRI images resulted in a 128 x 128 matrix (instead of the 92 x 92 imaging matrix), spatial smoothing (Gaussian filter of 1.4 voxel) was added. Because electrical VTA stimulation occasionally caused head motion, fMRI data sets with motion corrections of >1 mm in the x, y, z plane or 1° rotation between two consecutive frames were discarded from subsequent analysis. Functional activation was first analyzed by a linear regression analysis [general linear model (GLM) implemented in the *BrainVoyager QX*]. This means that signal intensity changes in each voxel were correlated with the given stimulus protocol. Based on this setup, an appropriate activation map was generated. To account for the hemodynamic delay, the stimulus representing block design was modified by a double-gamma hemodynamic response function (onset: 0 s; time to response peak: 5 s; time to undershoot peak: 15 s). To exclude false-positive voxels, a false-discovery rate (FDR) with a q-value of 0.05 (which corresponds to a t-value >3 or *p* < 0.005) was used as the threshold. Second, a volume of interest (VOI) analysis was used to depict activation of individual brain regions during the experiments. For that, each individual functional imaging data set was aligned to a 3D standard rat brain using the 3D volume tool implemented in BrainVoyager QX 2.6.1 software. Individual VOIs, i.e., right and left hippocampus, right and left nucleus accumbens, right and left striatum, septum, prefrontal cortex region and VTA was marked in the 3D standard rat brain. The average BOLD time series of all voxels located in one VOI was then calculated for each individual animal using the volume-of-interest-analysis tool implemented in the BrainVoyager QX2.6.1 software. Each individual BOLD time series was normalized using the averaged BOLD signal intensity as 100%. All normalized BOLD time series were then averaged and depicted as mean BOLD time series ± SD.

For the comparison of region-specific hemodynamic response functions (HRF), event-related BOLD responses (i.e., only significantly activated voxels (GLM analysis) or all voxels (VOI analysis) in the analyzed region) were calculated by measuring the signal intensities starting at 6 frames (−12 s until 0 s) before stimulus onset (stimulus presentation was between 0 and 8 s, which corresponds to 4 frames) until 20 frames (8–48 s) after the stimulus ends. The averaged signal intensities within the appropriate area in the first five frames (−12 s until −2 s) were set to 100%. Because there was high variability in the BOLD responses during the first 3 stimulation trains, only stimulation trains 4–25 were considered for the calculation of the event-related BOLD response. To visualize the activation pattern during each of the three stimulation blocks, all fMRI datasets were aligned to a 3D standard rat brain using anatomical landmarks. These datasets were then analyzed further with a linear regression analysis (GLM multisubject analysis, implemented in the *BrainVoyager QX* software).

To assess functional connectivity between individual VOIs, the Pearson correlation coefficients between the averaged BOLD time series of each VOI was calculated.

### Statistical analysis

All data were reported as group averages ± SEM. For statistical comparison of values among individual experiments, the nonparametric Wilcoxon-signed rank test from the statistical program *WinSTAT* (Ver. 2012.1) was used. A minimal number of six animals per group were used to perform the Wilcoxon-signed rank test. A paired Student’s *t*-test was used to compare BOLD signal intensities within an experimental group. Differences were considered statistically significant when *p* < 0.05.

## Results

Electrical stimulation of the VTA results in a massive dopamine release in target regions of the dopaminergic mesolimbic pathway [6], which may affect signal processing in these regions for a long time (i.e., for longer than 1 min) and thus, the resulting BOLD response to subsequent stimulations. To minimize the putative confounding effect of VTA stimulation on subsequent stimulation trains, the stimulation protocol did not consist of randomly applied CA3, VTA, and CA3/VTA stimulations, but consisted of three consecutive blocks, each block containing identical stimulation trains (Fig 1).

### Costimulation of CA3 diminishes VTA-mediated BOLD responses in the prefrontal cortex/anterior cingulate cortex area (experiment 1)

**Fig 2:**
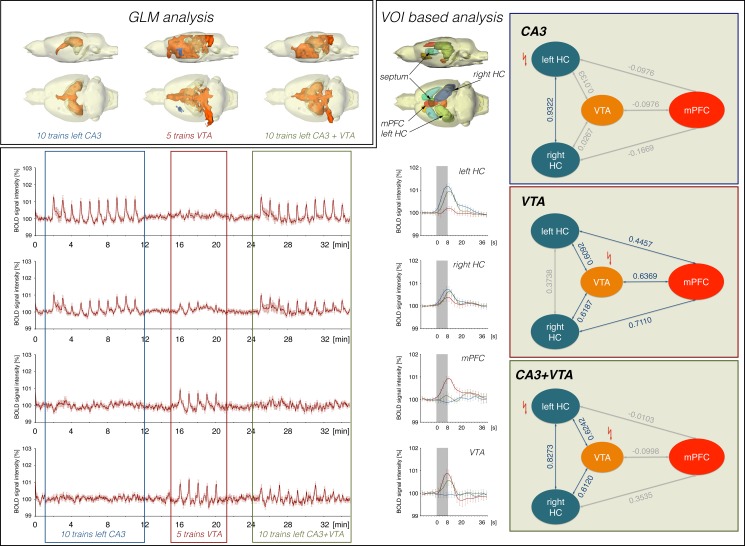
Costimulation of CA3 and VTA does not result in an additive BOLD response. (A) Distribution of significantly activated voxel during stimulation of the left CA3, VTA and subsequent costimulation of CA3 and VTA (threshold: fdr q = 0.001). (B) BOLD time series measured in individual brain regions (n = 11). (C) Location of analyzed VOIs and average BOLD responses for each stimulation condition in individual. (D) Summary of Pearson correlation coefficients describing the linear relationship between BOLD time series of the corresponding VOIs during each of the three stimulation conditions. Correlations (r > 0.4) are marked by solid arrows.

In the first set of experiments, the left CA3-region was stimulated with 10 consecutive stimulation trains followed by a stimulation of the VTA with 5 stimulation trains and finally, the two regions were simultaneously stimulated with 10 consecutive trains (n = 11). As previously observed [4], repetitive stimulation of the left CA3 region resulted in significant BOLD responses in the left and right hippocampus and the lateral and medial septal nuclei (Fig 2). At this low frequency, no significant changes in BOLD signal intensities were observed in the mPFC/ACC region. Subsequent electrical stimulations of the VTA induced significant BOLD responses in various regions along the midline of the brain, including the mPFC/ACC, septal nuclei and parts of the hippocampus (Fig 2), similar to previous results [6]. The subsequent costimulation of the left CA3 region and VTA caused in no region an additive BOLD response. Thus, BOLD responses in the left and right hippocampus only reached the level that was already observed during sole stimulation of the CA3 region during the first stimulation block. Similarly, the BOLD responses in the septal region did not sum up, although previous CA3 as well as VTA stimulation already caused significant BOLD responses in this area. In the mPFC/ACC region, costimulation of CA3 and VTA resulted in a significantly reduced BOLD response when compared with the BOLD responses induced during previous sole VTA stimulations. Thus, concurrent electrical stimulation of the CA3 region appears to inhibit VTA-mediated BOLD responses in the mPFC/ACC region.

**Fig 3:**
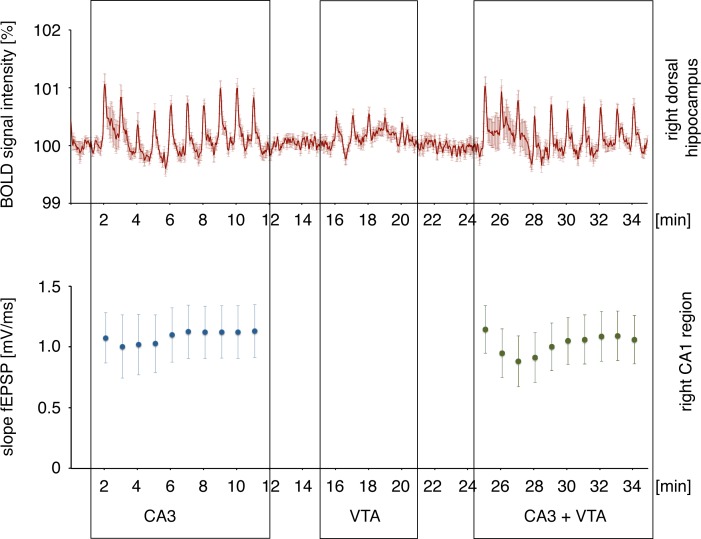
VTA stimulation does not significantly affect field potentials recorded in the right CA1 region during concurrent electrical stimulation of the left CA3. Top: BOLD time series (VOI analysis) of the right hippocampus. Bottom: concurrently measured field potentials in the right CA1 region. Depicted are the average responses of all 16 responses per train.

Electrophysiological responses in the right CA1 region that were induced by electrical stimulations of the left CA3 region were unaffected in the presence of concurrent electrical stimulations of the VTA during the last 10 stimulation trains; thus, putative short-term effects of VTA stimulations on CA1 responses could not be observed, at least on the level of postsynaptic CA1 pyramidal cell activation (Fig 3).

BOLD time series of individual brain regions (Fig 2, S1 Fig) were used to calculate functional connectivities that relate to concurrent BOLD signal intensity variations (S1 Table). As expected, electrical 2Hz pulse stimulation of the left CA3 only activates a small network comprising the left and right hippocampus and the septum. In contrast, electrical VTA stimulation caused the activation of a more widespread network, which includes the mPFC/ACC, septum and the left and right hippocampus. In addition, BOLD time series of the left and right striatum highly correlated although the BOLD time series of the two regions did not correlate with the BOLD time series in the VTA. Concurrent stimulation of right CA3 and VTA did not result in an additive network but in an apparent elimination of mPFC/ACC from the network (Fig 2).

### Consecutive VTA stimulation only causes moderate decreases in BOLD signals in the mPFC/ACC (experiment 2)

**Fig 4:**
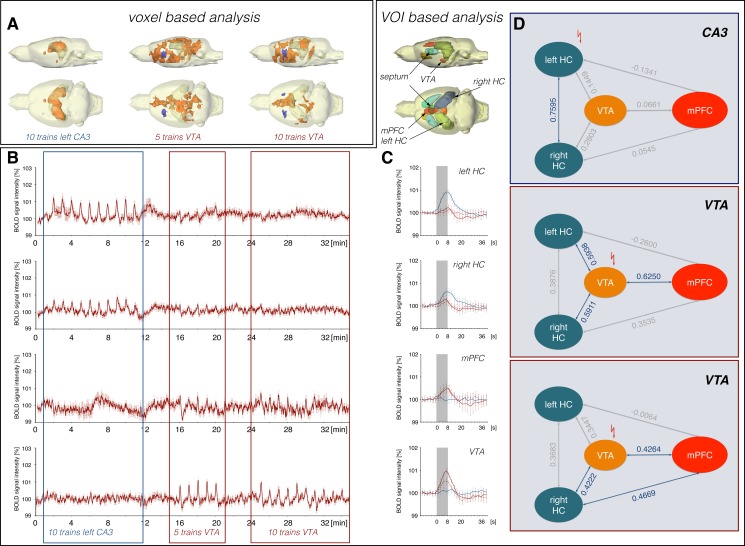
Repetitive electrical stimulations of the VTA do not inhibit BOLD responses in the mPFC/ACC. (A) Distribution of significantly activated voxels during CA3, initial, and late VTA stimulation (n = 9). (B) BOLD time series measured in individual brain regions (n = 11). (C) Average BOLD responses in individual VOIS. (D) Pearson correlation coefficients representing the linear relationships between BOLD time series in these VOIs. Color-coding similar to in Fig 2, dashed red line represents BOLD responses during the last 10 stimulation trains.

Significant decreases in BOLD responses during repetitive stimulations are frequently observed and may depend on factors other than specific inhibition. [2, 14, 15]. Therefore, we checked in a second set of experiments if the attenuation of BOLD responses in the mPFC/ACC region during the last 10 stimulation trains requires costimulation of the left CA3 region or only reflects adaptation of the hemodynamic response due to repetitive stimulations. The initial stimulation experiment was repeated, but only VTA stimulations were applied during the last 10 stimulation trains (n = 9, Figs 1 and 4). Under this condition, the formation of significant BOLD responses in the mPFC/ACC area and septum was not significantly reduced during the last 10 stimulation trains (Fig 4). Nevertheless, repetitive electrical stimulations of the VTA resulted in slightly decreasing significant BOLD responses in the VTA, which coincides with a reduced distribution of significantly activated voxels (Fig 4). This result supports the assumption that CA3 stimulation actually initiated the variation in BOLD response in the mPFC/ACC region during the last 10 stimulation trains.

Pearson correlations of BOLD time series from individual VOIs (S2 Fig) revealed similar network activities during initial stimulations of the right CA3 or VTA as observed during the first experiment (S2 Table). Sole VTA stimulations during the last 10 stimulation trains resulted in a similar network activity as observed during the initial VTA stimulations; in particular, there remained, although to a lesser degree, a correlation between VTA and mPFC/ACC BOLD time series (Fig 4).

### Repetition of electrical CA3 stimulation after a short interval causes similar BOLD responses (experiment 3)

**Fig 5:**
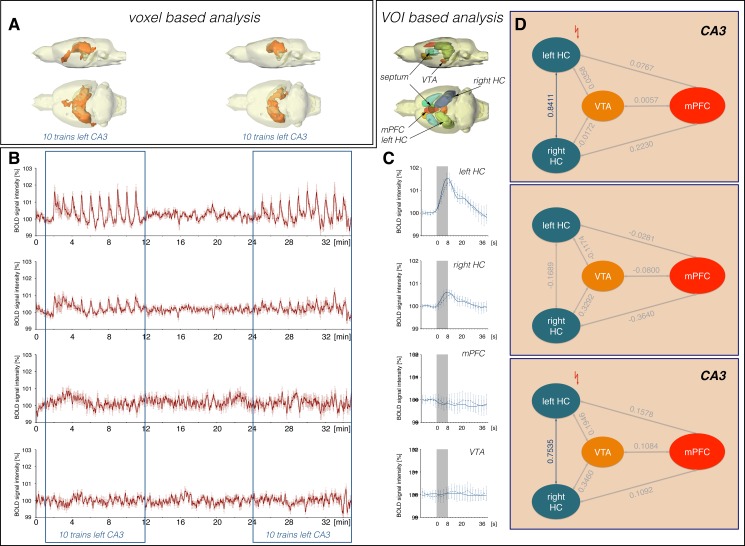
Repetitive electrical stimulations of the left CA3 do not affect BOLD responses in the mPFC/ACC. (A) Distribution of significantly activated voxels during two consecutive CA3 stimulation periods (n = 6). (B) Summary of BOLD time series in individual VOIs. (C) Average BOLD responses during initial CA3 (solid blue lines and late CA3 stimulations (dashed blue lines). (D) Pearson correlation coefficients representing the linear relationships between BOLD time series of two VOIs are summarized.

Frequent electrical stimulations of the hippocampal CA3 subregion may cause long-lasting effects on signal processing in the hippocampus and, as a consequence, an altered signal propagation to its target regions during later stimulations, in particular, after a short resting interval. To determine if the first 10 electrical CA3 stimulations would cause long-lasting changes in signal processing that would modify the formation of BOLD responses during the last 10 stimulation trains, we repeated the initial experiment without electrical VTA stimulations (n = 6, Figs 1 and 5). Under this condition, the BOLD responses in the left and right hippocampus developed similarly, i.e., during the last stimulation block, the magnitude of BOLD responses was almost similar. In the mPFC/ACC region, significantly activated voxels were neither observed during the first nor during the last 10 stimulation trains. Furthermore, no reduction of baseline BOLD signal intensities was observed during the last stimulation trains that could affect positive BOLD responses during additional activations of the VTA. Thus, repetitive electrical stimulations of the CA3 with continuous 2-Hz stimulation trains did not cause altered BOLD signal intensities in the mPFC region.

Pearson correlations of BOLD time series from individual VOIs (S3 Fig) reflect similar network activities during initial and late stimulations of the left CA3, except that during later trains, an increased correlation was found between the left and right striatum (Fig 5, S3 Table).

### Decrease in BOLD response is not a delayed action of VTA stimulation (experiment 4)

**Fig 6:**
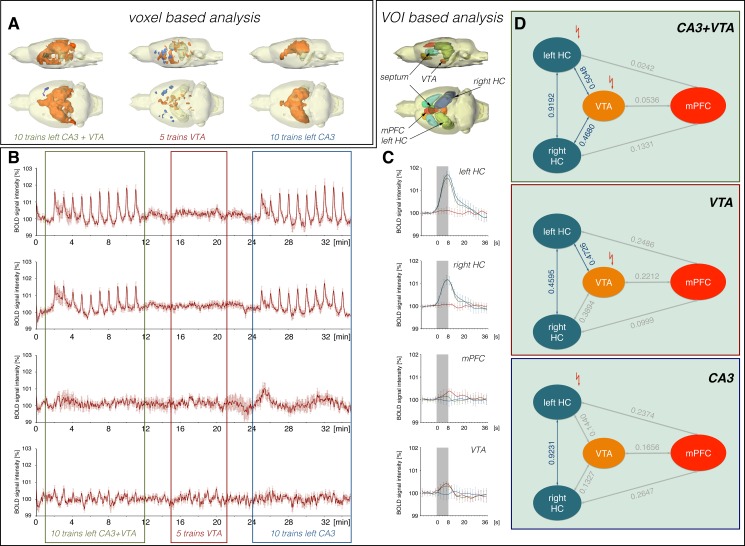
Electrical stimulation of the left CA3 suppressed VTA stimulation-induced BOLD responses in the mPFC/ACC. Concurrent electrical stimulations of the left CA3 and VTA during the initial 10 stimulation trains results in a similar BOLD response pattern as observed during the first experiment (see Fig 2) when this costimulation was applied during the last 10 stimulation trains (n = 8). Color coding of the graphs in this figure corresponds to the color coding in Fig 2.

In a fourth set of experiments, we checked if the initial individual stimulations of the CA3 and VTA region modified associated neuronal networks in such a way that subsequent concurrent stimulation of the two regions were no longer comparable with the initial stimulations. Therefore, we reversed the initial experiment, i.e., performed the costimulation of CA3 and VTA at the beginning (n = 8, Figs 1 and 6).

Costimulation of CA3 and VTA during the initial 10 stimulation trains caused a BOLD activation pattern that was similar to the pattern observed during the first experiment, in which the CA3/VTA costimulations were presented during the last 10 stimulation trains (Figs 2 and 6). In particular, BOLD responses in the mPFC/ACC were initially significantly smaller than during the following sole VTA stimulations. In contrast, an inhibitory effect of concurrent CA3 stimulation on the VTA stimulation-induced BOLD responses in the septum was not observed. There, the BOLD responses were similar during all three different stimulation conditions.

These results confirm the initial assumption that concurrent CA3 stimulation attenuates BOLD responses in the mPFC/ACC during VTA stimulation. In contrast, such an inhibitory effect on BOLD responses in the septum is not unambiguously observed.

Pearson correlations of BOLD time series from individual VOIs indicated a missing interaction between VTA and mPFC/ACC during the initial 10 stimulation trains and thus, an apparently similar network activity as observed during the first experiment under identical stimulation conditions during the last 10 stimulation trains (Fig 6, S4 Fig, S4 Table).

## Discussion

In the present study, the VTA and the left hippocampus were either separately or concurrently stimulated. These two structures project to common but also to different target regions; thus, electrical costimulation should result in a different activation pattern than the stimulation of only one structure. According to the simple assumption that functionally connected regions will display similar (i.e., highly correlated) BOLD time series, concurrent stimulations of the two structures should cause additive (or subtractive) BOLD responses in parts of common networks, whereas in parts of individual networks, the induced BOLD responses should match BOLD responses induced by stimulation of one single structure. In particular, an additive response should be expected when one target region concurrently receives direct and excitatory (glutamatergic) inputs from two structures. This is, evidently, not always the case. The main finding of the present study is that stimulation of the left hippocampus with low-frequency pulses (i.e., 2 Hz) results in an attenuation of BOLD responses in the mPFC/ACC during high frequency (i.e., bursts of 10 pulses at 100 Hz) VTA simulation. The mPFC receives direct glutamatergic, GABAergic, and dopaminergic projections from the VTA [16–19]. Among them, glutamatergic transmission controls the formation of significant BOLD responses in the mPFC/ACC during application of short bursts of high frequency pulses to the VTA, because presence of MK801, a NMDA receptor antagonist prevents the formation of significant BOLD responses in this region [6]. Similarly, the mPFC receives glutamatergic projections from hippocampal CA1 and subicular pyramidal cells [20, 21]. In contrast to high-frequency VTA stimulation, low frequency stimulation of the left CA3 region did not induce significant BOLD responses in the mPFC/ACC, a result that confirms two previous studies [4, 5]. Thus, putative variations in neuronal activities in the mPFC/ACC induced by hippocampal afferents were not sufficient to induce detectable hemodynamic responses; this requires pulse frequencies of 10 Hz or higher, which in turn caused the formation of positive BOLD responses in the mPFC [5]. However, the activity induced by 2 Hz pulse stimulation of the left CA3 was sufficient to reduce BOLD responses induced by afferent fibers from the VTA.

There are basically two general scenarios that conceive of how hippocampal activity inhibits VTA-mediated BOLD responses in the mPFC. *First*, hippocampal activity could result in an inhibition of glutamatergic VTA neurons, which in turn leads to reduced activation of the mPFC. There are several facts that disagree with this scenario. (I) VTA stimulation occurred before CA3 stimulation (Fig 1), (II) one stimulation electrode was located in the VTA; thus, electrical stimulation directly activates glutamatergic VTA neurons, and (III) concurrently induced effects in the septum and striatum were not always affected by additional CA3 stimulations (Fig 6). *Second*, during low frequency (i.e., 2 Hz) CA3 stimulation hippocampal activity will be propagated to the mPFC where, although it will not affect the local hemodynamic by itself, it modifies the induced activation pattern elicited by incoming activity from the VTA. This could happen either at the level of neuronal activity or at the level of neuro-vascular coupling. As mentioned above, hippocampal projections to the mPFC are mainly glutamatergic, whereas VTA projections to the mPFC are dopaminergic, GABAergic and glutamatergic [16]. In a previous, similar study, we found that BOLD responses in the mPFC/ACC that were induced by the same electrical VTA stimulation protocol were almost completely abolished in the presence of MK801, an NMDA receptor antagonist but not by SCH23390, a D_1/5_ dopamine receptor antagonist. Furthermore, selective activation of dopaminergic neurons in the VTA by an optogenetic approach resulted in no significant BOLD responses in the mPFC/ACC [6]. Thus the formation of significant BOLD responses in the mPFC/ACC during electrical VTA stimulation mainly depends on glutamatergic transmission [6] although dopaminergic neurons may also affect the hemodynamic response [22–24], however at a much lower level. So far, it is not known if glutamatergic afferents from the hippocampus and from the VTA target the same or a different subset of neurons in the mPFC/ACC. In any case, if the magnitude of the BOLD response in the mPFC/ACC were only related to the incoming (glutamatergic) activity, a reduced BOLD response during costimulation of the two afferent systems would be virtually impossible. Glutamatergic fibers from the hippocampus target not only excitatory pyramidal cells but also inhibitory interneurons within the mPFC [25]. Thus, activation of inhibitory interneurons by hippocampal afferents may reduce subsequent activation by glutamatergic VTA afferents, reflected in the reduced BOLD response. Future, more detailed electrophysiological studies have to be performed to study the interaction between glutamatergic hippocampal and VTA afferents in the mPFC.

Animal studies about functional connectivities are often confounded by the presence of anesthetics or sedatives, which in order to be effective modify the efficacy of certain transmitter systems. This is a particular concern when functional connectivities are analyzed under resting condition, i.e., when no external stimuli are presented and local BOLD signal fluctuations only reflect endogenous neuronal activities [26]. In the present study, electrical stimulation of one or two defined anatomical regions was used to reveal the corresponding target regions. Thus, functional connectivities, e.g., between VTA and mPFC, were visualized by the application of electrical pulses to the VTA, which in turn activated projecting neurons in the VTA and subsequently targeted neurons in the mPFC. Although medetomidine, the used sedative, reduces glutamatergic transmission via activation of presynaptic alpha-2 adrenergic receptors it did not prevent stimulus-induced activation of the mPFC. Thus under the here used experimental condition the anatomical connectivity between VTA and mPFC remained functional under the presence of medetomidine, but functional different during costimulation of the hippocampal CA3 region.

The unexpected finding that an additional incoming excitatory glutamatergic input diminishes the formation of a BOLD response in the mPFC/ACC has substantial implications for the interpretation of apparent functional connectivities that are determined by correlating BOLD signal intensity changes in various brain regions. In the experimental approach used, functional connectivity (in terms of neurophysiological interactions) between VTA and mPFC/ACC remained similar during the entire first experiment (Fig 2) because direct electrical stimulation of (glutamatergic) VTA neurons causatively drove this functional connectivity. However, functional connectivity (in terms of temporal correlation between spatially individual VOI BOLD time series) only depends on synchronized local BOLD signal variations, and it only assumes that this synchronicity is caused by neurophysiological interactions of these structures. Our results now indicate that strong interactions (i.e., functional connectivities) may still be present under certain circumstances but remain undetectable by BOLD fMRI analysis. In other words, conclusions about functional connectivities based on BOLD-fMRI are not unambiguous, and the detection of altered functional connectivities by BOLD-fMRI may reflect rather an altered activity of a modulatory system than an actual altered existing neurophysiological interaction, especially under conditions that are sometimes barely verifiable (e.g., attentions, vigilance, sensory inputs, emotions). In particular, under specific pathological conditions, still existing actual functional connectivities between two particular regions (in terms of neurophysiological interactions) may be missed because confounding disease-related otherwise altered activity patterns modify the computed functional connectivities (in terms of correlated BOLD signal intensity changes). That means that the underlying pathological processes are not always necessarily identical to apparent changes in functional connectivities between two regions as detected by BOLD-fMRI; the cause might even be localized in other remote regions. Therefore, for an unambiguous interpretation of altered functional connectivities under pathological conditions in humans, an fMRI approach should be combined with additional studies, preferentially with imaging modalities that directly measure neurophysiological parameters of regional interactions.

## Supporting information

S1 Fig BOLD time series measured in individual brain regions during experiment 1. Average BOLD responses (i.e., for significantly activated voxels in the particular region) for each stimulation condition are summarized at the right side (blue graphs: CA3 stimulation, red graphs: VTA stimulation, green graphs: CA3 and VTA stimulation).
(TIF)

S2 Fig BOLD time series measured in individual brain regions during experiment 2. Average BOLD responses (i.e., for significantly activated voxels in the particular region) for each stimulation condition are summarized at the right side (blue graphs: CA3 stimulation, solid red graphs: initial VTA stimulation period, dashed red graphs: second VTA stimulation period).
(TIF)

S3 Fig BOLD time series measured in individual brain regions during experiment 3. Average BOLD responses (i.e., for significantly activated voxels in the particular region) for each stimulation condition are summarized at the right side (solid blue graphs: initial CA3 stimulation period, dashed blue graphs: second CA3 stimulation period).
(TIF)

S4 Fig BOLD time series measured in individual brain regions during experiment 4. Average BOLD responses (i.e., for significantly activated voxels in the particular region) for each stimulation condition are summarized at the right side (blue graphs: CA3 stimulation, red graphs: VTA stimulation, green graphs: CA3 and VTA stimulation).
(TIF)

S1 Table Pearson correlation coefficients calculated from BOLD time series of analyzed VOIs measured during experiment 1. (see also Fig 2, S1 Fig).
(DOCX)

S2 Table Pearson correlation coefficients calculated from BOLD time series of analyzed VOIs measured during experiment 2. (see also Fig 4, S2 Fig).
(DOCX)

S3 Table Pearson correlation coefficients calculated from BOLD time series of analyzed VOIs measured during experiment 3. (see also Fig 5, S3 Fig).
(DOCX)

S4 Table Pearson correlation coefficients calculated from BOLD time series of analyzed VOIs measured during experiment 4. (see also Fig 6, S4 Fig).
(DOCX)

## References

[1] Angenstein F, Kammerer E, Scheich H. The BOLD Response in the Rat Hippocampus Depends Rather on Local Processing of Signals than on the Input or Output Activity. A Combined Functional MRI and Electrophysiological Study. J Neurosci. 2009;29(8):2428–39. 10.1523/JNEUROSCI.5015-08.2009 19244518 PMC6666263

[2] Angenstein F, Krautwald K, Scheich H. The current functional state of local neuronal circuits controls the magnitude of a BOLD response to incoming stimuli. Neuroimage. 2010;50(4):1364–75. 10.1016/j.neuroimage.2010.01.070 20114080

[3] Logothetis NK. The neural basis of the blood-oxygen-level-dependent functional magnetic resonance imaging signal. Philos Trans R Soc Lond B Biol Sci. 2002;357(1424):1003–37. 10.1098/rstb.2002.1114 12217171 PMC1693017

[4] Scherf T, Angenstein F. Postsynaptic and spiking activity of pyramidal cells, the principal neurons in the rat hippocampal CA1 region, does not control the resultant BOLD response: a combined electrophysiologic and fMRI approach. J Cereb Blood Flow Metab. 2015;35(4):565–75. 10.1038/jcbfm.2014.252 25564229 PMC4420892

[5] Moreno A, Morris RG, Canals S. Frequency-Dependent Gating of Hippocampal-Neocortical Interactions. Cereb Cortex. 2015. 10.1093/cercor/bhv033 25761637

[6] Helbing C, Brocka M, Scherf T, Lippert MT, Angenstein F. The role of the mesolimbic dopamine system in the formation of blood-oxygen-level dependent responses in the medial prefrontal/anterior cingulate cortex during high-frequency stimulation of the rat perforant pathway. J Cereb Blood Flow Metab. 2016;36(12):2177–93. 10.1177/0271678X15615535 26661229 PMC5363663

[7] Lisman JE, Grace AA. The hippocampal-VTA loop: controlling the entry of information into long-term memory. Neuron. 2005;46(5):703–13. 10.1016/j.neuron.2005.05.002 15924857

[8] Roitman MF, Stuber GD, Phillips PE, Wightman RM, Carelli RM. Dopamine operates as a subsecond modulator of food seeking. J Neurosci. 2004;24(6):1265–71. 10.1523/JNEUROSCI.3823-03.2004 14960596 PMC6730321

[9] Schmidt HD, Famous KR, Pierce RC. The limbic circuitry underlying cocaine seeking encompasses the PPTg/LDT. Eur J Neurosci. 2009;30(7):1358–69. PubMed Central PMCID: PMC2875792. 10.1111/j.1460-9568.2009.06904.x 19788581 PMC2875792

[10] Steinberg EE, Boivin JR, Saunders BT, Witten IB, Deisseroth K, Janak PH. Positive reinforcement mediated by midbrain dopamine neurons requires D1 and D2 receptor activation in the nucleus accumbens. PLoS ONE. 2014;9(4):e94771 PubMed Central PMCID: PMC3986242. 10.1371/journal.pone.0094771 24733061 PMC3986242

[11] Paxinos G, Watson C. The Rat Brain in Stereotaxic coordinates. San Diego: Academic Press; 1988.

[12] Weber R, Ramos-Cabrer P, Wiedermann D, van Camp N, Hoehn M. A fully noninvasive and robust experimental protocol for longitudinal fMRI studies in the rat. Neuroimage. 2006;29(4):1303–10. 10.1016/j.neuroimage.2005.08.028 16223588

[13] Hennig J, Nauerth A, Friedburg H. RARE imaging: a fast imaging method for clinical MR. Magn Reson Med. 1986;3:823–33. 3821461 10.1002/mrm.1910030602

[14] Angenstein F. The actual intrinsic excitability of granular cells determines the ruling neurovascular coupling mechanism in the rat dentate gyrus. J Neurosci. 2014;34(25):8529–45. 10.1523/JNEUROSCI.0472-14.2014 24948808 PMC6608210

[15] Riemann S, Helbing C, Angenstein F. From default to adjusted, how the BOLD response in the rat hippocampus develops during consecutive stimulations. J Cereb Blood Flow Metab. 2016; 10.1177/0271678X16634715 PMC5381453 26911895

[16] Fields HL, Hjelmstad GO, Margolis EB, Nicola SM. Ventral tegmental area neurons in learned appetitive behavior and positive reinforcement. Annu Rev Neurosci. 2007;30:289–316. 10.1146/annurev.neuro.30.051606.094341 17376009

[17] Lavin A, Nogueira L, Lapish CC, Wightman RM, Phillips PE, Seamans JK. Mesocortical dopamine neurons operate in distinct temporal domains using multimodal signaling. J Neurosci. 2005;25(20):5013–23. 10.1523/JNEUROSCI.0557-05.2005 15901782 PMC5509062

[18] Morales M, Root DH. Glutamate neurons within the midbrain dopamine regions. Neuroscience. 2014;282:60–8. 10.1016/j.neuroscience.2014.05.032 24875175 PMC4397110

[19] Taylor SR, Badurek S, Dileone RJ, Nashmi R, Minichiello L, Picciotto MR. GABAergic and glutamatergic efferents of the mouse ventral tegmental area. J Comp Neurol. 2014;522(14):3308–34. PubMed Central PMCID: PMCPMC4107038. 10.1002/cne.23603 24715505 PMC4107038

[20] Amaral D, Lavenex P. Hippocampal Neuroanatomy In: Andersen Per, Morris Richard, Amaral David, Bliss Tim, O'Keefe J, editors. The Hippocampus Book. Oxford, New York: Oxford University Press; 2007 p. 37–114.

[21] Thierry AM, Gioanni Y, Degenetais E, Glowinski J. Hippocampo-prefrontal cortex pathway: anatomical and electrophysiological characteristics. Hippocampus. 2000;10(4):411–9. 10.1002/1098-1063(2000)10:4<411::AID-HIPO7>3.0.CO;2-A 10985280

[22] Decot HK, Namboodiri VM, Gao W, McHenry JA, Jennings JH, Lee SH, et al Coordination of Brain Wide Activity Dynamics by Dopaminergic Neurons. Neuropsychopharmacology. 2016. 10.1038/npp.2016.151 PMC5240174 27515791

[23] Ferenczi EA, Zalocusky KA, Liston C, Grosenick L, Warden MR, Amatya D, et al Prefrontal cortical regulation of brainwide circuit dynamics and reward-related behavior. Science. 2016;351(6268):aac9698 PubMed Central PMCID: PMCPMC4772156. 10.1126/science.aac9698 26722001 PMC4772156

[24] Lohani S, Poplawsky AJ, Kim SG, Moghaddam B. Unexpected global impact of VTA dopamine neuron activation as measured by opto-fMRI. Mol Psychiatry. 2016. 10.1038/mp.2016.102 PMC5269559 27457809

[25] Parent MA, Wang L, Su J, Netoff T, Yuan LL. Identification of the hippocampal input to medial prefrontal cortex in vitro. Cereb Cortex. 2010;20(2):393–403. PubMed Central PMCID: PMCPMC2803736. 10.1093/cercor/bhp108 19515741 PMC2803736

[26] Grandjean J, Schroeter A, Batata I, Rudin M. Optimization of anesthesia protocol for resting-state fMRI in mice based on differential effects of anesthetics on functional connectivity patterns. Neuroimage. 2014;102 Pt 2:838–47. 25175535 10.1016/j.neuroimage.2014.08.043

