# Supplementary figures and images for: Hippocampal CA3 activation alleviates fMRI-BOLD responses in the rat prefrontal cortex induced by electrical VTA stimulation
Source: PLoS One. 2017 Feb 27;12(2):e0172926. doi: 10.1371/journal.pone.0172926 (PMC5328285; doi:10.1371/journal.pone.0172926)

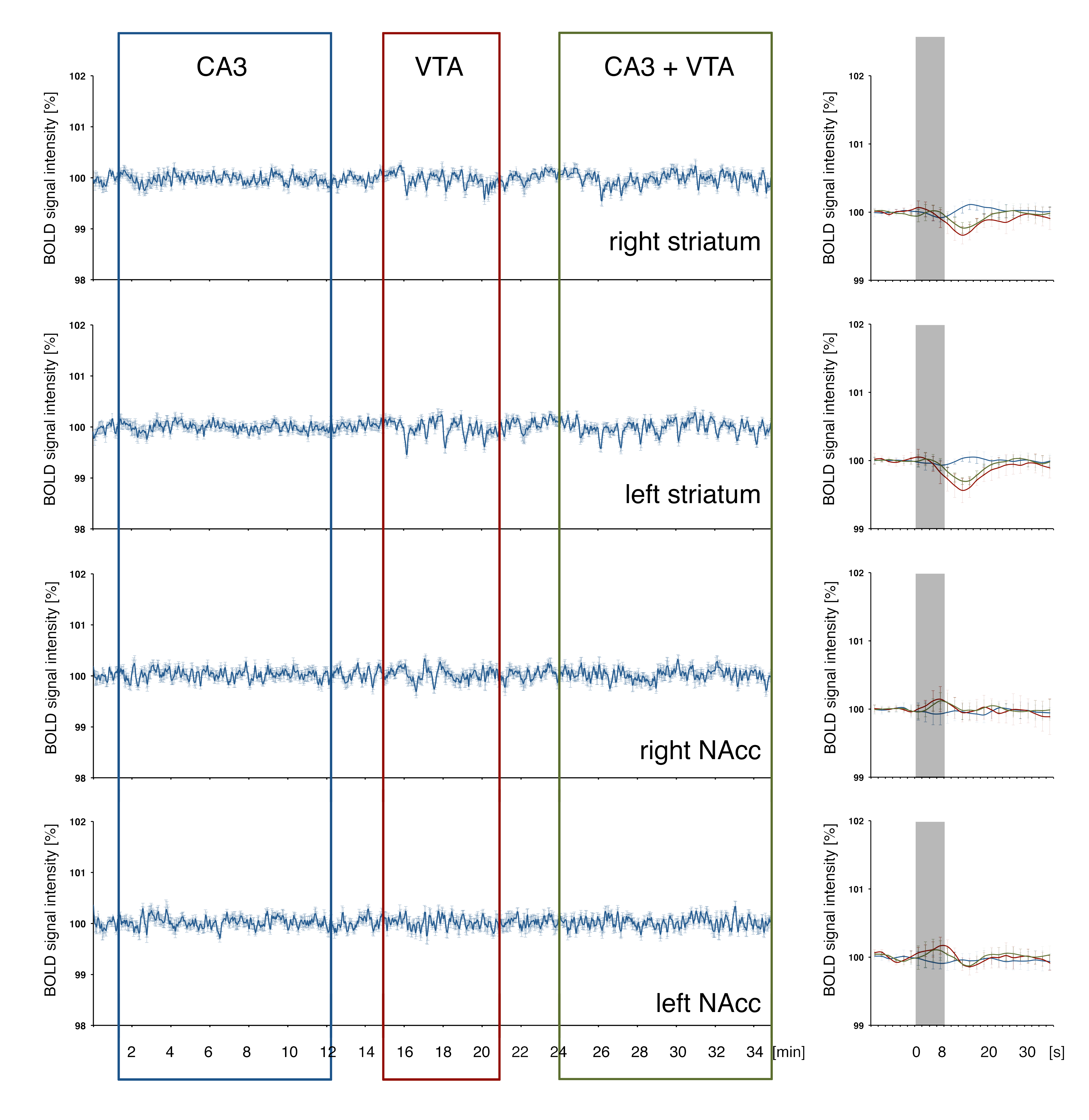

Supplement: S1 Fig — Average BOLD responses (i.e., for significantly activated voxels in the particular region) for each stimulation condition are summarized at the right side (blue graphs: CA3 stimulation, red graphs: VTA stimulation, green graphs: CA3 and VTA stimulation). (TIF) [file pone.0172926.s001.tif]

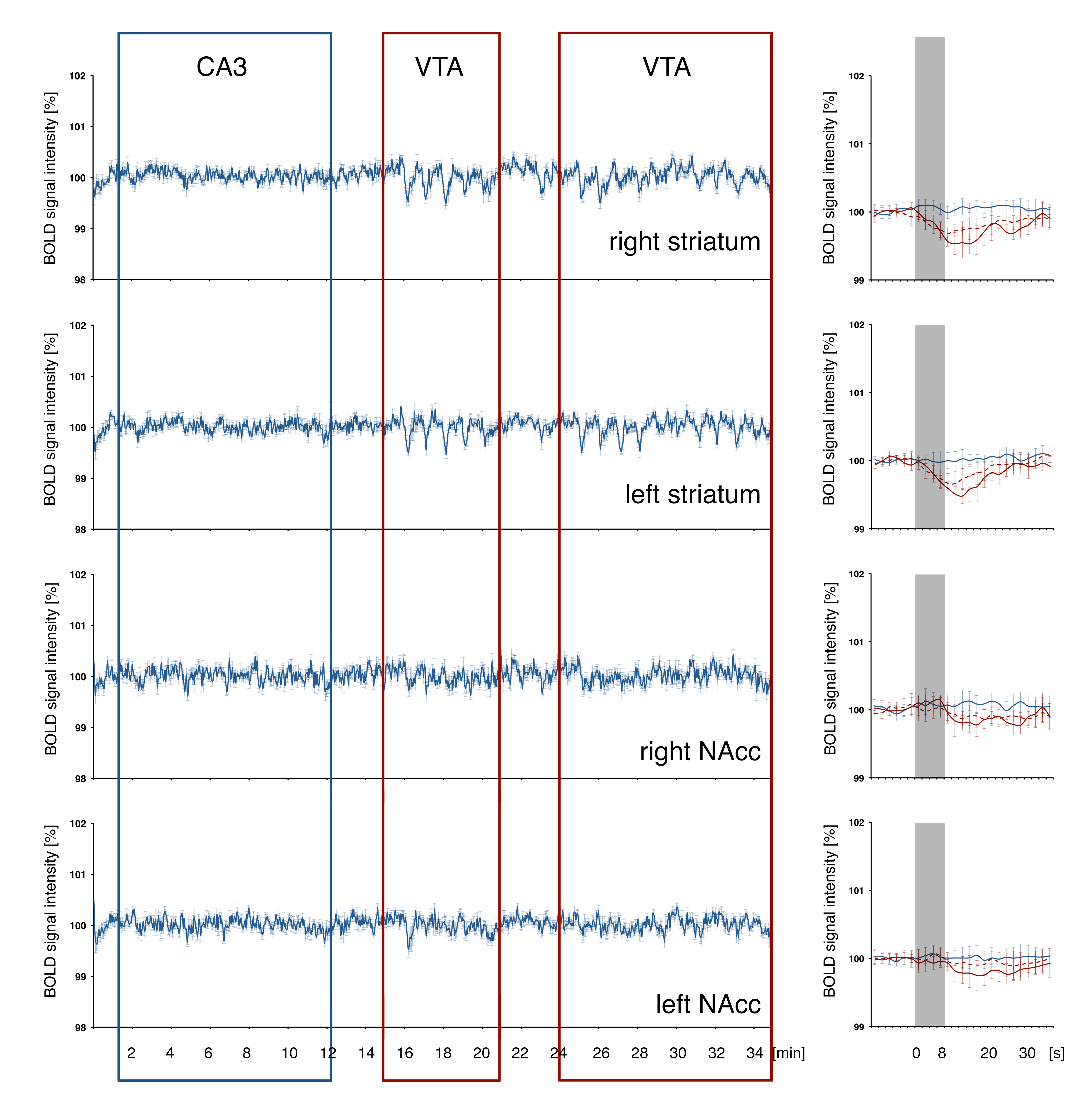

Supplement: S2 Fig — Average BOLD responses (i.e., for significantly activated voxels in the particular region) for each stimulation condition are summarized at the right side (blue graphs: CA3 stimulation, solid red graphs: initial VTA stimulation period, dashed red graphs: second VTA stimulation period). (TIF) [file pone.0172926.s002.tif]

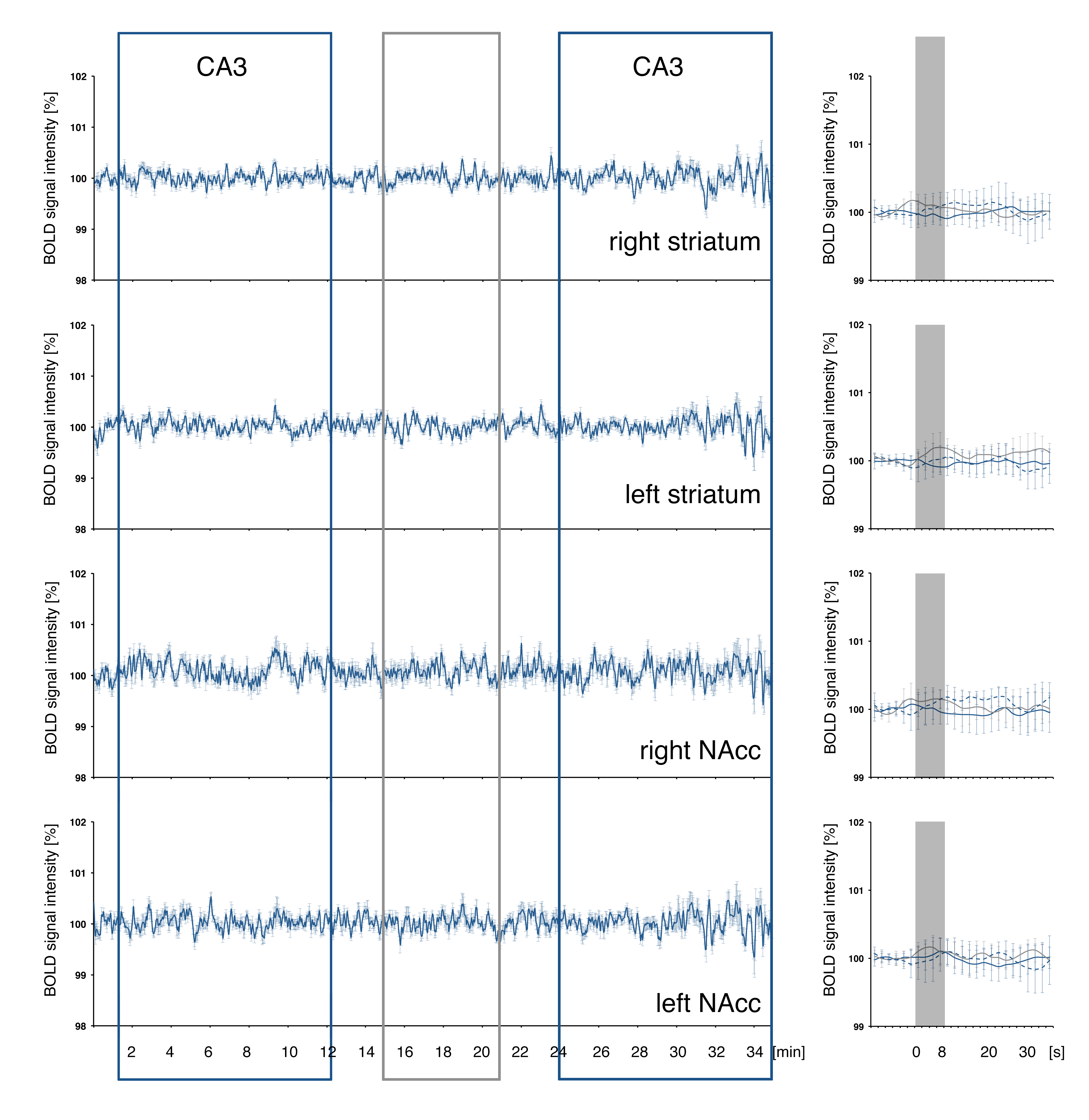

Supplement: S3 Fig — Average BOLD responses (i.e., for significantly activated voxels in the particular region) for each stimulation condition are summarized at the right side (solid blue graphs: initial CA3 stimulation period, dashed blue graphs: second CA3 stimulation period). (TIF) [file pone.0172926.s003.tif]

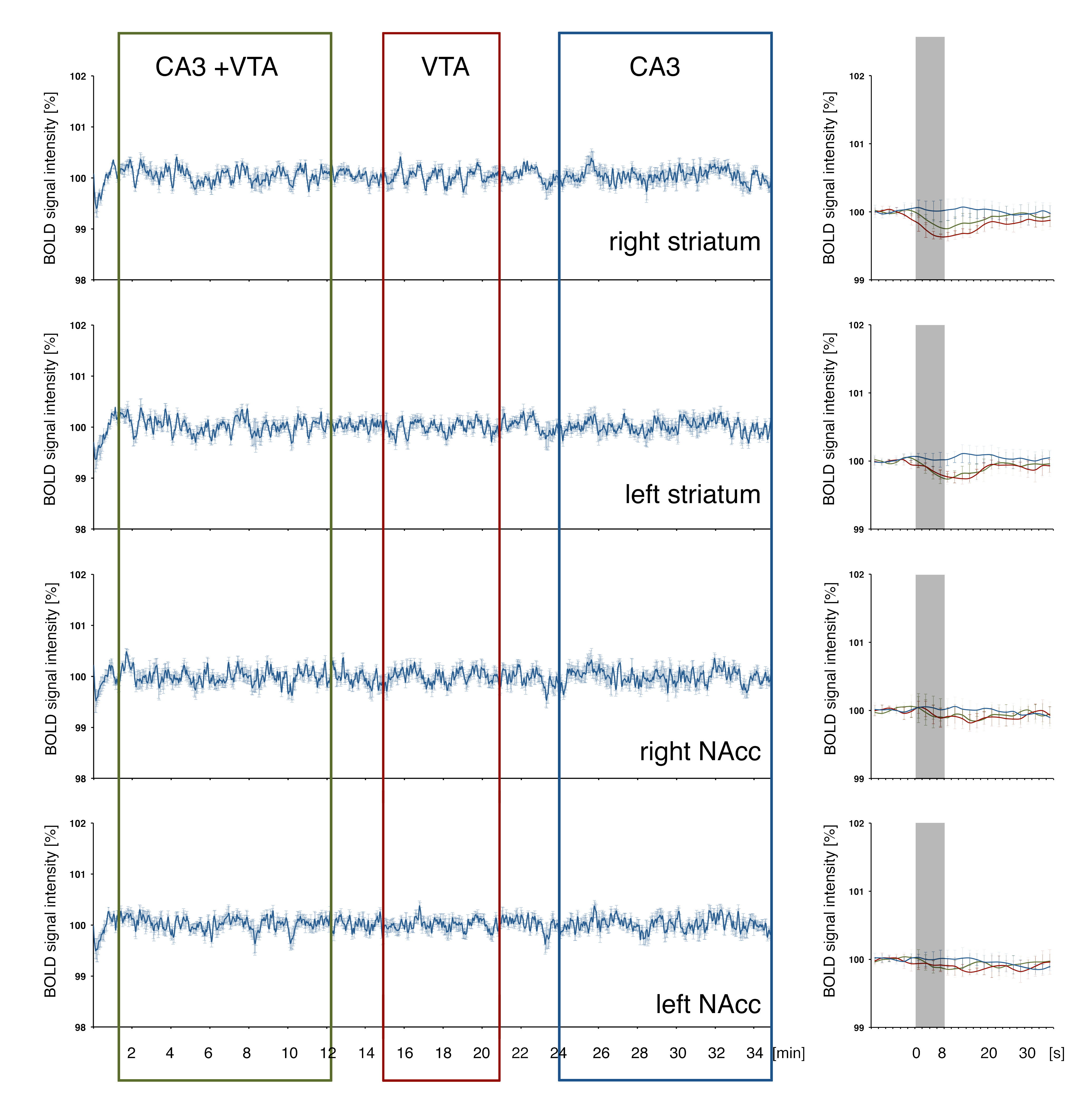

Supplement: S4 Fig — Average BOLD responses (i.e., for significantly activated voxels in the particular region) for each stimulation condition are summarized at the right side (blue graphs: CA3 stimulation, red graphs: VTA stimulation, green graphs: CA3 and VTA stimulation). (TIF) [file pone.0172926.s004.tif]
