# Supplementary material for: Hippocampal CA3 activation alleviates fMRI-BOLD responses in the rat prefrontal cortex induced by electrical VTA stimulation
Source: PLoS One. 2017 Feb 27;12(2):e0172926. doi: 10.1371/journal.pone.0172926 (PMC5328285; doi:10.1371/journal.pone.0172926)
Supplement: S1 Table — (see also Fig 2, S1 Fig). (DOCX) [file pone.0172926.s005.docx]

|  |  |  |  |  |  |  |  |  |  |  |
| --- | --- | --- | --- | --- | --- | --- | --- | --- | --- | --- |
| \| **1 CA3** \| le-HC \| ri HC \| mPFC \| ri NAcc \| septum \| VTA \| le stria \| ri stria \| le NAcc \| \| --- \| --- \| --- \| --- \| --- \| --- \| --- \| --- \| --- \| --- \| \| le-HC \| 1 \|  \|  \|  \|  \|  \|  \|  \|  \| \| ri HC \| **0.9323** \| 1 \|  \|  \|  \|  \|  \|  \|  \| \| mPFC \| -0.2776 \| -0.1669 \| 1 \|  \|  \|  \|  \|  \|  \| \| ri NAcc \| 0.0801 \| 0.0594 \| 0.0782 \| 1 \|  \|  \|  \|  \|  \| \| septum \| **0.8252** \| **0.8187** \| -0.1475 \| 0.0381 \| 1 \|  \|  \|  \|  \| \| VTA \| 0.0133 \| 0.0267 \| -0.0976 \| 0.0118 \| 0.0716 \| 1 \|  \|  \|  \| \| le stria \| -0.1599 \| -0.2082 \| 0.0717 \| 0.1496 \| -0.0570 \| -0.0379 \| 1 \|  \|  \| \| ri stria \| -0.2102 \| -0.2381 \| 0.0247 \| 0.0928 \| -0.0835 \| -0.0335 \| **0.4825** \| 1 \|  \| \| le NAcc \| -0.0564 \| -0.0131 \| 0.2544 \| 0.1913 \| 0.0272 \| 0.2313 \| 0.1941 \| 0.1639 \| 1 \| \|  \|  \|  \|  \|  \|  \|  \|  \|  \|  \| \|  \|  \|  \|  \|  \|  \|  \|  \|  \|  \| \| **2 VTA** \| le-HC \| ri HC \| mPFC \| ri NAcc \| septum \| VTA \| le stria \| ri stria \| le NAcc \| \| le-HC \| 1 \|  \|  \|  \|  \|  \|  \|  \|  \| \| ri HC \| 0.3738 \| 1 \|  \|  \|  \|  \|  \|  \|  \| \| mPFC \| **0.4457** \| 0.7110 \| 1 \|  \|  \|  \|  \|  \|  \| \| ri NAcc \| **0.4871** \| 0.2902 \| 0.3803 \| 1 \|  \|  \|  \|  \|  \| \| septum \| **0.5512** \| **0.6975** \| **0.8637** \| 0.3727 \| 1 \|  \|  \|  \|  \| \| VTA \| **0.6092** \| **0.6187** \| **0.6369** \| 0.3762 \| **0.7706** \| 1 \|  \|  \|  \| \| le stria \| -0.1797 \| -0.2438 \| **-0.5318** \| 0.0693 \| **-0.4879** \| -0.1970 \| 1 \|  \|  \| \| ri stria \| -0.1816 \| -0.2661 \| -0.3694 \| 0.1213 \| -0.3760 \| -0.1471 \| **0.7976** \| 1 \|  \| \| le NAcc \| 0.3212 \| 0.2910 \| 0.3304 \| **0.4138** \| 0.2955 \| 0.3960 \| 0.3444 \| 0.3317 \| 1 \| \|  \|  \|  \|  \|  \|  \|  \|  \|  \|  \| \|  \|  \|  \|  \|  \|  \|  \|  \|  \|  \| \| **3 CA3+VTA** \| le-HC \| ri HC \| mPFC \| ri NAcc \| septum \| VTA \| le stria \| ri stria \| le NAcc \| \| le-HC \| 1 \|  \|  \|  \|  \|  \|  \|  \|  \| \| ri HC \| **0.8273** \| 1 \|  \|  \|  \|  \|  \|  \|  \| \| mPFC \| -0.0103 \| 0.3535 \| 1 \|  \|  \|  \|  \|  \|  \| \| ri NAcc \| 0.3879 \| 0.3209 \| 0.0198 \| 1 \|  \|  \|  \|  \|  \| \| septum \| **0.7873** \| **0.7915** \| 0.1893 \| 0.2464 \| 1 \|  \|  \|  \|  \| \| VTA \| **0.6242** \| **0.6120** \| 0.0998 \| 0.2300 \| **0.6621** \| 1 \|  \|  \|  \| \| le stria \| **-0.4112** \| **-0.4994** \| -0.1282 \| 0.0202 \| **-0.5159** \| -0.1423 \| 1 \|  \|  \| \| ri stria \| -0.1989 \| -0.3591 \| -0.1808 \| 0.2448 \| -0.3820 \| -0.0987 \| **0.6917** \| 1 \|  \| \| le NAcc \| 0.0334 \| 0.0746 \| 0.1549 \| 0.0521 \| -0.0531 \| 0.1028 \| 0.2894 \| 0.2097 \| 1 \| |  |  |  |  |  |  |  |  |  |  |
|  |  |  |  |  |  |  |  |  |  |  |
|  |  |  |  |  |  |  |  |  |  |  |
|  |  |  |  |  |  |  |  |  |  |  |
| **S1 Table.** **Pearson correlation coefficients calculated from BOLD time series of analyzed VOIs measured during experiment 1** (see also Fig 2, S1 Fig). |  |  |  |  |  |  |  |  |  |  |
|  |  |  |  |  |  |  |  |  |  |  |
