# Supplementary material for: Hippocampal CA3 activation alleviates fMRI-BOLD responses in the rat prefrontal cortex induced by electrical VTA stimulation
Source: PLoS One. 2017 Feb 27;12(2):e0172926. doi: 10.1371/journal.pone.0172926 (PMC5328285; doi:10.1371/journal.pone.0172926)
Supplement: S2 Table — (see also Fig 4, S2 Fig). (DOCX) [file pone.0172926.s006.docx]

| **1 CA3** | le-HC | ri HC | mPFC | ri NAcc | septum | VTA | le stria | ri stria | le NAcc |
| --- | --- | --- | --- | --- | --- | --- | --- | --- | --- |
| le-HC | 1 |  |  |  |  |  |  |  |  |
| ri HC | **0.7595** | 1 |  |  |  |  |  |  |  |
| mPFC | -0.1341 | 0.0545 | 1 |  |  |  |  |  |  |
| ri NAcc | 0.1271 | 0.0995 | 0.0619 | 1 |  |  |  |  |  |
| septum | **0.5665** | **0.6522** | 0.0982 | 0.1802 | 1 |  |  |  |  |
| VTA | 0.1449 | 0.2803 | 0.0661 | 0.0650 | 0.2688 | 1 |  |  |  |
| le stria | -0.0613 | 0.0342 | 0.0104 | 0.1695 | 0.0758 | 0.1191 | 1 |  |  |
| ri stria | 0.0904 | 0.0781 | -0.0123 | 0.3043 | 0.0996 | 0.0264 | 0.2480 | 1 |  |
| le NAcc | 0.0675 | 0.2101 | 0.2387 | 0.2087 | 0.2776 | 0.1882 | 0.2210 | 0.1687 | 1 |
|  |  |  |  |  |  |  |  |  |  |
|  |  |  |  |  |  |  |  |  |  |
| **2 VTA** | le-HC | ri HC | mPFC | ri NAcc | septum | VTA | le stria | ri stria | le NAcc |
| le-HC | 1 |  |  |  |  |  |  |  |  |
| ri HC | 0.3876 | 1 |  |  |  |  |  |  |  |
| mPFC | 0.2600 | 0.5609 | 1 |  |  |  |  |  |  |
| ri NAcc | 0.1799 | 0.4753 | 0.3630 | 1 |  |  |  |  |  |
| septum | **0.4173** | **0.6010** | **0.7214** | **0.4421** | 1 |  |  |  |  |
| VTA | **0.5938** | **0.5911** | **0.6250** | 0.2502 | **0.7772** | 1 |  |  |  |
| le stria | -0.0203 | 0.0929 | -0.0206 | 0.3168 | -0.1962 | -0.2870 | 1 |  |  |
| ri stria | 0.0598 | 0.3035 | 0.0736 | **0.5076** | -0.0485 | -0.1938 | **0.7335** | 1 |  |
| le NAcc | 0.1540 | **0.4039** | **0.5086** | **0.4030** | 0.3023 | 0.2179 | **0.4992** | **0.4790** | 1 |
|  |  |  |  |  |  |  |  |  |  |
|  |  |  |  |  |  |  |  |  |  |
| **3 VTA** | le-HC | ri HC | mPFC | ri NAcc | septum | VTA | le stria | ri stria | le NAcc |
| le-HC | 1 |  |  |  |  |  |  |  |  |
| ri HC | 0.3683 | 1 |  |  |  |  |  |  |  |
| mPFC | -0.0064 | 0.4669 | 1 |  |  |  |  |  |  |
| ri NAcc | 0.3053 | **0.4004** | 0.1897 | 1 |  |  |  |  |  |
| septum | 0.1185 | **0.4039** | **0.5265** | 0.3559 | 1 |  |  |  |  |
| VTA | 0.3447 | **0.4222** | **0.4264** | 0.3320 | **0.6890** | 1 |  |  |  |
| le stria | 0.1398 | 0.0960 | -0.2953 | 0.3417 | -0.2518 | -0.2545 | 1 |  |  |
| ri stria | 0.3032 | 0.1974 | -0.2637 | **0.4219** | -0.1374 | -0.0547 | **0.6493** | 1 |  |
| le NAcc | 0.2094 | 0.3409 | 0.1235 | **0.4385** | 0.2654 | 0.2437 | 0.3989 | 0.3978 | 1 |

**S2 Table.** **Pearson correlation coefficients calculated from BOLD time series of analyzed VOIs measured during experiment 2** (see also Fig 4, S2 Fig).
