# Supplementary material for: Hippocampal CA3 activation alleviates fMRI-BOLD responses in the rat prefrontal cortex induced by electrical VTA stimulation
Source: PLoS One. 2017 Feb 27;12(2):e0172926. doi: 10.1371/journal.pone.0172926 (PMC5328285; doi:10.1371/journal.pone.0172926)
Supplement: S3 Table — (see also Fig 5, S3 Fig). (DOCX) [file pone.0172926.s007.docx]

| **1 CA3** | le-HC | ri HC | mPFC | ri NAcc | septum | VTA | le stria | ri stria | le NAcc |
| --- | --- | --- | --- | --- | --- | --- | --- | --- | --- |
| le-HC | 1 |  |  |  |  |  |  |  |  |
| ri HC | **0.8411** | 1 |  |  |  |  |  |  |  |
| mPFC | 0.0767 | 0.2230 | 1 |  |  |  |  |  |  |
| ri NAcc | 0.1514 | 0.1939 | 0.3415 | 1 |  |  |  |  |  |
| septum | **0.6241** | **0.6051** | 0.3620 | 0.3495 | 1 |  |  |  |  |
| VTA | 0.0358 | -0.0172 | 0.0057 | 0.0691 | 0.0268 | 1 |  |  |  |
| le stria | -0.1202 | -0.1241 | 0.0598 | 0.2369 | -0.1253 | 0.1258 | 1 |  |  |
| ri stria | -0.0818 | 0.0319 | -0.0170 | 0.2883 | 0.0161 | -0.1596 | 0.2116 | 1 |  |
| le NAcc | 0.1348 | 0.1877 | 0.2980 | 0.2904 | 0.1779 | 0.1211 | 0.2659 | 0.1314 | 1 |
|  |  |  |  |  |  |  |  |  |  |
|  |  |  |  |  |  |  |  |  |  |
| **2 no** | le-HC | ri HC | mPFC | ri NAcc | septum | VTA | le stria | ri stria | le NAcc |
| le-HC | 1 |  |  |  |  |  |  |  |  |
| ri HC | -0.1689 | 1 |  |  |  |  |  |  |  |
| mPFC | -0.0281 | -0.3640 | 1 |  |  |  |  |  |  |
| ri NAcc | 0.2455 | -0.0343 | -0.1297 | 1 |  |  |  |  |  |
| septum | 0.1857 | -0.1335 | 0.1115 | 0.2794 | 1 |  |  |  |  |
| VTA | -0.1174 | 0.3292 | -0.0800 | -0.0420 | -0.1672 | 1 |  |  |  |
| le stria | 0.2816 | 0.1317 | -0.1804 | 0.1906 | -0.1589 | 0.0480 | 1 |  |  |
| ri stria | 0.0620 | -0.0561 | -0.1203 | 0.5760 | 0.2638 | 0.0221 | 0.0348 | 1 |  |
| le NAcc | 0.2052 | -0.0928 | -0.1153 | 0.1917 | 0.2859 | 0.0135 | -0.0844 | 0.0778 | 1 |
|  |  |  |  |  |  |  |  |  |  |
|  |  |  |  |  |  |  |  |  |  |
| **3 CA3** | le-HC | ri HC | mPFC | ri NAcc | septum | VTA | le stria | ri stria | le NAcc |
| le-HC | 1 |  |  |  |  |  |  |  |  |
| ri HC | **0.7535** | 1 |  |  |  |  |  |  |  |
| mPFC | 0.1578 | 0.1092 | 1 |  |  |  |  |  |  |
| ri NAcc | 0.2649 | 0.3725 | 0.2934 | 1 |  |  |  |  |  |
| septum | **0.4507** | **0.5433** | **0.4120** | **0.4940** | 1 |  |  |  |  |
| VTA | 0.1946 | 0.3460 | 0.1084 | 0.3222 | 0.3151 | 1 |  |  |  |
| le stria | 0.3109 | 0.4747 | 0.3015 | 0.4145 | 0.4319 | **0.4441** | 1 |  |  |
| ri stria | 0.3973 | 0.4459 | 0.3150 | 0.6638 | 0.4295 | 0.3132 | **0.5462** | 1 |  |
| le NAcc | 0.2246 | 0.3045 | 0.2315 | 0.4674 | 0.3148 | **0.4954** | **0.6894** | **0.5216** | 1 |

**S3 Table. Pearson correlation coefficients calculated from BOLD time series of analyzed VOIs measured during experiment 3** (see also Fig 5, S3 Fig).
