# Supplementary material for: Hippocampal CA3 activation alleviates fMRI-BOLD responses in the rat prefrontal cortex induced by electrical VTA stimulation
Source: PLoS One. 2017 Feb 27;12(2):e0172926. doi: 10.1371/journal.pone.0172926 (PMC5328285; doi:10.1371/journal.pone.0172926)
Supplement: S4 Table — (see also Fig 6, S4 Fig). (DOCX) [file pone.0172926.s008.docx]

| **1 CA3+VTA** | le-HC | ri HC | mPFC | ri NAcc | septum | VTA | le stria | ri stria | le NAcc |
| --- | --- | --- | --- | --- | --- | --- | --- | --- | --- |
| le-HC | 1 |  |  |  |  |  |  |  |  |
| ri HC | **0.9192** | 1 |  |  |  |  |  |  |  |
| mPFC | 0.0242 | 0.1331 | 1 |  |  |  |  |  |  |
| ri NAcc | -0.0887 | -0.0326 | 0.0128 | 1 |  |  |  |  |  |
| septum | **0.7193** | **0.6976** | -0.0081 | 0.0774 | 1 |  |  |  |  |
| VTA | **0.5048** | **0.4680** | 0.0536 | 0.2535 | **0.5951** | 1 |  |  |  |
| le stria | **-0.4015** | -0.2411 | 0.1615 | **0.4002** | -0.1800 | -0.0679 | 1 |  |  |
| ri stria | -0.3345 | -0.1767 | 0.2299 | **0.4450** | -0.1118 | -0.0289 | **0.7476** | 1 |  |
| le NAcc | -0.2085 | -0.0770 | 0.0683 | **0.4358** | -0.0292 | 0.0684 | **0.5599** | **0.5539** | 1 |
|  |  |  |  |  |  |  |  |  |  |
|  |  |  |  |  |  |  |  |  |  |
| **2 VTA** | le-HC | ri HC | mPFC | ri NAcc | septum | VTA | le stria | ri stria | le NAcc |
| le-HC | 1 |  |  |  |  |  |  |  |  |
| ri HC | **0.4595** | 1 |  |  |  |  |  |  |  |
| mPFC | 0.2486 | 0.0999 | 1 |  |  |  |  |  |  |
| ri NAcc | **0.4513** | 0.1497 | -0.0867 | 1 |  |  |  |  |  |
| septum | 0.3542 | 0.2400 | 0.3857 | 0.2075 | 1 |  |  |  |  |
| VTA | **0.4726** | 0.3894 | 0.2212 | 0.3183 | **0.4492** | 1 |  |  |  |
| le stria | 0.1888 | 0.2115 | -0.2060 | 0.3209 | 0.1832 | 0.1015 | 1 |  |  |
| ri stria | 0.0685 | 0.0796 | -0.3417 | **0.4075** | -0.1429 | -0.1370 | **0.5816** | 1 |  |
| le NAcc | 0.2793 | 0.2361 | -0.1569 | **0.4852** | 0.0767 | 0.2679 | **0.4665** | **0.4351** | 1 |
|  |  |  |  |  |  |  |  |  |  |
|  |  |  |  |  |  |  |  |  |  |
| **3 CA3** | le-HC | ri HC | mPFC | ri NAcc | septum | VTA | le stria | ri stria | le NAcc |
| le-HC | 1 |  |  |  |  |  |  |  |  |
| ri HC | **0.9231** | 1 |  |  |  |  |  |  |  |
| mPFC | 0.1374 | 0.2647 | 1 |  |  |  |  |  |  |
| ri NAcc | 0.3366 | 0.3443 | 0.4445 | 1 |  |  |  |  |  |
| septum | **0.6816** | **0.7570** | 0.3299 | **0.4184** | 1 |  |  |  |  |
| VTA | 0.1440 | 0.1327 | 0.1656 | 0.2917 | 0.2226 | 1 |  |  |  |
| le stria | 0.1710 | 0.2570 | **0.4860** | **0.4557** | 0.3632 | 0.2724 | 1 |  |  |
| ri stria | 0.2475 | 0.3322 | 0.5621 | 0.5753 | 0.3578 | 0.2583 | **0.5675** | 1 |  |
| le NAcc | 0.2185 | 0.3297 | 0.2832 | 0.3101 | **0.4327** | 0.2492 | 0.3571 | 0.3547 | 1 |

**S4 Table.** **Pearson correlation coefficients calculated from BOLD time series of analyzed VOIs measured during experiment 4** (see also Fig 6, S4 Fig).
